# The first records of *Stenobermuda* Schultz, 1982 and *Tenupedunculus* Schultz, 1979 from Australia, with description of two new species from the Great Barrier Reef (Isopoda, Asellota, Stenetriidae)

**DOI:** 10.3897/zookeys.733.20474

**Published:** 2018-01-26

**Authors:** Ji-Hun Song, Niel L. Bruce, Gi-Sik Min

**Affiliations:** 1 Department of Biological Sciences, Inha University, 100 Inha-ro, Nam-gu, Incheon 22212, South Korea; 2 Museum of Tropical Queensland, Queensland Museum, 70-102 Flinders Street, Townsville, Australia 4810; 3 Unit for Environmental Sciences and Management Water Research Group (Ecology), North West University, Potchefstroom 2520, South Africa

**Keywords:** Asellota, Australia, Great Barrier Reef, new species, Stenetriidae, *Stenobermuda*, *Tenupedunculus*

## Abstract

The genera *Tenupedunculus* Schultz, 1982 and *Stenobermuda* Schultz, 1979 are recorded for the first time from beyond the Southern Ocean, at the Great Barrier Reef, Australia. *Tenupedunculus serrulatus* **sp. n.** and *Stenobermuda warooga* **sp. n.** are described from Heron Island and Lizard Island respectively, both in the Great Barrier Reef. The genus *Tenupedunculus* is revised and a new diagnosis presented, with *Tenupedunculus virginale* Schultz, 1982, *T. pulchrum* (Schultz, 1982), and *T. serrulatus* **sp. n.** being retained within the genus, and the remaining species here regarded as Stenetriidae *incertae sedis* (eight species).

## Introduction

The family Stenetriidae Hansen, 1905 comprises 12 accepted genera (Wilson and Schotte 2008). Serov and Wilson (1995) provided the only comprehensive generic review and reappraisal of this family. More recently the genera *Machatrium* Bruce & Buxton, 2013 and *Onychatrium* Bruce & Cumming, 2015 were described, including new species from the Great Barrier Reef, Australia. Marine stenetriids are diverse, abundant and omni-present on coral reefs (Kensley 1984a; b; 1988; Müller 1990; 1991a, b; Kensley and Schotte 2002; Martin et al. 2003), but still remains relatively poorly documented in tropical Australia (see Bruce and Cumming 2015).

In this paper, two new species of Stenetriidae are described from Great Barrier Reef, Australia: *Tenupedunculus serrulatus* sp. n. and *Stenobermuda warooga* sp. n. These are the first records of these genera from Australian waters. The genus *Stenobermuda* is known to occur on coral reefs in East Africa (Kensley and Schotte 2002) and this is the first record of the genus from the western Pacific. *Tenupedunculus* has hitherto included deep-water Southern Ocean species, often incompletely described, and presenting an inconsistent suite of characters at the generic level. *Tenupedunculus* is here revised and a restrictive diagnosis presented. *Tenupedunculus serrulatus* is the first record of the genus from shallow water and from coral reefs.

Including the present genera the Great Barrier Reef is now known to have four genera of Stenetriidae. The rich collections made during the Census of Marine Life’s (CoML) Census of Coral Reef Ecosystems (CReefs) Program, housed at the Museum of Topical Queensland, hold abundant specimens of the genera *Liocoryphe* Serov & Wilson, 1995, *Tristenium* Serov & Wilson, 1995, *Mizothenar* Serov & Wilson, 1995 and *Stenetrium* Haswell, 1881 (from Ningaloo Reef, Western Australia).

## Materials and methods

**Sampling.** See Bruce (2015) and Bruce and Buxton (2013) for details of sampling methods and locations.

**Descriptions.** See Bruce and Buxton (2013) for a detailed account of pereopod morphology. Descriptions were generated using a DELTA database (Dallwitz et al. 2006; Coleman et al. 2010). Whole animals were drawn using a stereomicroscope (Leica MZ125, Wetzlar, Germany) and dissected appendages were drawn using a light microscope (Leica DM2500, Wetzlar, Germany) equipped with differential interference contrast and a camera lucida. Dissected appendages were temporarily mounted on slides using an 85% lactic acid solution, lightly stained with lignin pink.

**Digital inking.** Pencil illustrations were scanned and electronically inked using a graphics tablet (Wacom Intuos4, Düsseldorf, Germany) and Adobe Illustrator CS5. A dorsal view of the pleotelson for each species was drawn with the aspect specifically positioned to allow for descriptive measurements. In habitus drawings, specimen curvature of these animals often distorts the true length of the pleotelson.

**Permits**. Specimens were collected under permits: Great Barrier Reef Maine Park Authority GBRMPA G08/26156.1, G09/32313.1; Queensland Fisheries Service QFS 95152.

**Abbreviations.** MTQ – Museum of Tropical Queensland; RS – robust seta/e.

## Taxonomy

### Suborder Asellota Latreille, 1802

#### Family Stenetriidae Hansen, 1905

##### Genus Tenupedunculus

Taxon classificationAnimaliaIsopodaStenetriidae

Schultz, 1982 sensu stricto

Tenupedunculus Schultz, 1982: 77.– Serov and Wilson 1995: 77.

###### Type species.

*Tenupedunculus elongatus* Schultz, 1982; by original designation and monotypy.

###### Species included.

*Tenupedunculus elongatus* (type species), south-eastern Argentine Basin, 4696 m; *T. virginale* (Schultz, 1982), Scotia Sea, Antarctica, 567 m; *T. pulchrum* (Schultz, 1982), southern Argentina, 1911 m; *T. serrulatus* sp. n., Great Barrier Reef, Australia, 25 m.

Species here excluded from *Tenupedunculus* *s. str.*, are regarded as **Stenetriidae*incertae sedis***: *Tenupedunculus acutum* (Vanhöffen, 1914); *T. beddardi* (Kussakin, 1967); *T. dentimanum* (Kussakin, 1967); *T. drakensis* (Schultz, 1982); *T. inflectofrons* (Schultz, 1982); *T. serraticaudum* (Kussakin & Vasina, 1984); *T. smirnovi* (Vasina, 1982); and *T. haswelli* (Beddard, 1886).

###### Diagnosis

**(male).** Cephalon frontal margin antennal spines small; lateral spines moderate, acute, slightly longer than antennal spines or sub-equal length. Pseudorostrum quadrate to trapezoid, wider than long. Eyes small, round. Male pereopod 1 ischium–carpus superodistal margin produced with acute process, inferodistal margins not produced; propodus moderate, length 1.2–1.5 times maximum width, 1.9–2.1 times carpus length, propodal palm transversely truncate or distally inflected; dactylus length similar to propodus distal width. Male pleopod 2 appendix masculina bluntly rounded apically, without apical setae.

###### Description

**(male).** *Body* dorsal surface smooth or sparsely setose, widest at pereonites 6 and 7; *pereonite 1* length greater than 0.9 times pereonite 2 length; *pereonites 2–4* lateral margins convex, anteriorly acute. *Pleotelson* length subequal to width; lateral margins or finely serrate, sub-parallel, posterolateral spines prominent, margin posterior to spines rounded with weak or no apical lobe; dorsal surface smooth, or sparsely setose. *Cephalon* lateral margins smooth or finely serrate. *Antennae* length equal or longer than total body length, article 1 lateral spine absent. *Pereopod 1* basis superior margin with irregularly spaced setae along length; propodal palm with teeth along palm margin; dactylus length subequal to propodal palm length. *Pleopod 1* protopod rectangular, lateral margin setae present; rami lateral margins evenly convex. *Pleopod 2* protopod longer than wide, distal apex blunt, transversely truncate; *appendix masculina* lateral margin groove absent. *Pleopod 5* distal apex with 3–5 plumose setae.

###### Remarks.

The genus *Tenupedunculus* Schultz, 1982 was established as a monotypic genus based on a single male specimen lacking legs. Serov and Wilson (1995), who included eleven species in the genus, doubted the unity (= monophyly) of *Tenupedunculus* without referring to specific characters. Our overview of the species included in *Tenupedunculus* recognizes that many species are inadequately described, some lacking details of any pereopods or of the male pereopods and some lacking description of the male pleopods 1 and 2. There are two significant characters that differ between the species formerly placed in *Tenupedunculus* that we consider to be of generic significance, namely eye shape and pseudorostrum shape. Those species with large reniform eyes (or traces thereof) and an elongate pseudorostrum are here all excluded from *Tenupedunculus* Schultz, 1982 *sensu stricto.*

The characters that serve best to identify the genus *Tenupedunculus* are: small round eyes; anterior margin of head with only lateral spines prominent; pseudorostrum wider than long, quadrate or trapezoid; inferodistal margin of ischium–carpus in male pereopod 1 without process, and superodistal margin of ischium–carpus usually produced and acute (strongly produced as a process in *T. serrulatus* sp. n.). The principle differentiating and diagnostic characters for *Tenupedunculus* *sensu stricto* are presented in Table 1.

**Table 1.** Principle differentiating characters for *Tenupedunculus* *sensu stricto* (male).

	*Tenupedunculus* *sensu stricto*	‘*dentimanum* group’
1. Eyes	small, round	large, reniform
2. Cephalon, lateral spines	moderate	large
3. Pseudorostrum	quadrate to trapezoid, wider than long	round to acute, longer than wide
4. Pereopod 1 ischium–carpus superodistal margin	produced, with acute process	produced, with acute process(except carpus)
5. Appendix masculina	without apical setae	with apical setae
6. Appendix masculina	bluntly rounded apically	excavate rounded apically

###### Distribution.

All species of the genus, with the exception of the new species, are from the Antarctic and the sub-Antarctic region—off Argentina, Patagonian Shelf and also Scotia Sea (all Atlantic sector); at depths 500 to 4696 metres. *Tenupedunculus serrulatus* sp. n., from the southern Great Barrier Reef, is the first record of the genus from beyond the Southern Ocean and from depths less than 500 metres.

###### Remarks on the species excluded from *Tenupedunculus*.

The species listed here are retained in *Tenupedunculus* Schultz, 1982, but excluded from the genus *sensu stricto* as they either lack the diagnostic characters of *Tenupedunculus* or possess unique characters that also preclude their inclusion in other stenetriid genera. The here termed ‘*dentimanum* group’ of species, particularly when considered in conjunction with their shared characters, potentially warrants a new genus.

‘***dentimanum* group**’

All species share the following characters: cephalon antennal spines small, lateral spines large. Pseudorostrum anteriorly round to acute, as long as or longer than wide. Eyes large, reniform. Male pereopod 1 ischium and merus superodistal margins weakly to strongly forming an acute process, inferodistal margins not produced. Male pleopod 2 appendix masculina excavate, apically rounded, with apical setae.

*Tenupedunculus beddardi* (Kussakin, 1967). Southern Argentina; 680 m; similar to *T. dentimanum* with the following characters common to the group: pseudorostrum approximately as long as wide, lateral spines large, eyes reniform and male pereopod 1 carpus with distinct acute superodistal process. The pseudorostrum uniquely converges to a narrowly rounded apex. The appendix masculina is acute (not excavate), differing significantly from that of others in this group, but on balance the species otherwise agrees well with and is best placed within the ‘*dentimanum* group’ at present.

*Tenupedunculus dentimanum* (Kussakin, 1967). Southern Argentina; 680 m; pseudorostrum as long as posterior width, anteriorly broadly rounded.

*Tenupedunculus inflectofrons* (Schultz, 1982). Scotia Sea, Antarctica; 588 m; pseudorostrum rounded; male pereopod 1 not known; appendix masculina with terminal process.

*Tenupedunculus smirnovi* (Vasina, 1982). Patagonian Shelf; 500 m. Female only; seems to have reniform eyes, pseudorostrum stepped, acute; female pereopod 1 with ischium and merus with acute superodistal margin but not carpus; pleotelson with distinct caudomedial lobe.

**Ungrouped species.**

*Tenupedunculus acutum* (Vanhöffen, 1914). Gauss Station, Davis Sea; 3397 m; pseudorostrum longer than wide, anteriorly rounded with median point; moderate lateral spines on cephalon; eyes moderate in size (more than six ommatidia) round (eye shape is not entirely clear in the original figures); appendix masculina blunt (excavate), with apical setae; male pereopod 1 ischium and merus with acute processes but carpus without process. Eye size and shape precludes inclusion of *T. acutum* in the ‘*dentimanum* group’.

*Tenupedunculus haswelli* (Beddard, 1886). Rio del la Plata; 1097 m; eyes reniform; male pereopod 1 with superodistal process on carpus (i.e. pereopod 1 similar to *Tenupedunculus serrulatus* sp. n.); not evident if there is a rostrum or pseudorostrum; eyes reniform; appendix masculina not known.

*Tenupedunculus drakensis* (Schultz, 1982). Tierra del Fuego, Argentina; 548 m; pseudorostrum rounded to acute; weak lateral spines on cephalon; reniform eyes; appendix masculina not known; male pereopod 1 not known. Originally placed in *Protallocoxa* Schultz, 1978 this species was later transferred to *Tenupedunculus* by Serov and Wilson (1995).

*Tenupedunculus serraticaudum* (Kussakin & Vasina, 1984). South Atlantic; 500 m; pseudorostrum anteriorly round to acute; large lateral spines on cephalon; reniform eyes; appendix masculina blunt (excavate) with apical setae; male pereopod 1 carpus with superodistal process.

###### Key to the species of *Tenupedunculus* *sensu stricto*

**Table:** 

1	Lateral margins of the body (from cephalon to pleotelson) with serrations; shallow-water species, found at depths less than 50 m	***T. serrulatus* sp. n.**
–	Lateral margins of the body (from cephalon to pleotelson) without serrations; deep-water species, found at depths greater than 500 m	**2**
2	Posterior margin of pleotelson distinctly produced	***T. virginale***
–	Posterior margin of pleotelson not produced, obtusely or evenly rounded	**3**
3	Posterior margin of pleotelson obtusely rounded, with indications of uropodal bases	***T. pulchrum***
–	Posterior margin of pleotelson evenly rounded, without indications of uropodal bases	***T. elongatus***

##### Tenupedunculus serrulatus

Taxon classificationAnimaliaIsopodaStenetriidae

Song & Bruce sp. n.

http://zoobank.org/EB46C41A-6548-4BBA-9C12-1B39042FF916

Figs 1 , 2 , 3 , 4

###### Material examined.

All material from Capricorn Group, southern Great Barrier Reef.

###### Holotype.

♂ (4.2 mm), ‘Harry’s Bommie, Heron Island, 23.46053°S, 151.9293°E, 13 November 2010, reef slope, dead *Acropora*, 9 m, CReefs stn. HI10-002C, coll. C. Buxton (MTQ W33638).

###### Paratypes.

2 ♂ (4.5, 4.1 mm [dissected]), same sample as holotype, (MTQ W52903). ♀ (5.8 mm [pereopod 1 dissected]), same data as holotype (MTQ W33654). ♂ (5.1 mm [pereopod 7 dissected]), Sykes Reef west, 23.4316°S, 152.0493°E, 14 November 2010, reef slope, 27 m, CReefs stn. HI10-009F, coll. J. Reimer (MTQ W33694). 5 ♀ (3.2–5.0 mm), 1 juv. (1.5 mm), Sykes Reef west, 23.4316°S, 152.0493°E, 14 November 2010, reef slope, 27 m, CReefs stn. HI10-009F, coll. J. Reimer (MTQ W33695). 20 ♂ and ♀, same data as holotype, coll. C. Buxton, stn. HI10-002B (MTQ W33673, W33644) and HI10-002C (MTQ W33636, W33642). ♀ (3.5 mm), Lamont Reef, 23.5932°S, 152.0655°E, 16 November 2010, reef slope, dead *Acropora*, 9 m, CReefs stn. HI10-019B/1, coll. M. Blazewicz (MTQ W33753). 5 ♂ (3.8–5.8 mm), Heron Island, southern side ‘Twin Peaks’, 23°28.357'S, 151°57.593'E, 28 November 2009, small rubble, 13–17 m CReefs stn. HI09-125F, coll. N.L. Bruce & K. Schnabel. (MTQ W52904). ♀ (5.8 mm), Heron Island 23.43238°S, 152.03375°E, 14 November 2009, CReefs stn. 018, no other data (MTQ W52905). ♂ (3.5 mm), 2 ♀ (2.8, 5.1 mm), Lamont Reef, southern side, 23°36.125'S, 152°03.152'E, 19 November 2009, coarse sand and small rubble, 9.7 m, CReefs stn. HI09-058D, coll. K. Schnabel & N.L. Bruce. (MTQ exW31591). MTQ W52906). 8 ♂ and ♀, Harry’s Canyons, Heron Reef, 23°28.389'S, 151°57.835'E, 18 November 2009, reef slope, small rubble and coarse sand, 6 m, CReefs stn HI09-045D, coll. N.L. Bruce & K. Schnabel (MTQ W52907). 2 ♂, 2 ♀, 2 imm., Sykes Reef, 23°25.929'S, 152°02.924'E, 18 November 2009, 26 m, coll. S. Smith & A. Anderson (MTQ W52908).

###### Non-type.

All Heron Island: north-eastern side, 20 November 2009, small rubble and sand at base of large bommies 7 m, CReefs stn HI09-064D (MTQ W31595). “The Patches” (=Mystery Bommie), 28 November 2009, rubble, mid-channel, 18 m, CReefs stn HI09-123C (MTQ W31604). “Harrys Bommie”, 13 November 2010, dead coral on sandy bottom, CReefs stn HI10-002B, 10 m (MTQ W33669).

###### Etymology.

From combining the Latin words ‘*serrula*’ (serrated) and the ending of ‘*marginatus*’, alluding to the serrated body margins of this species.

###### Diagnosis

**Figure 1:**
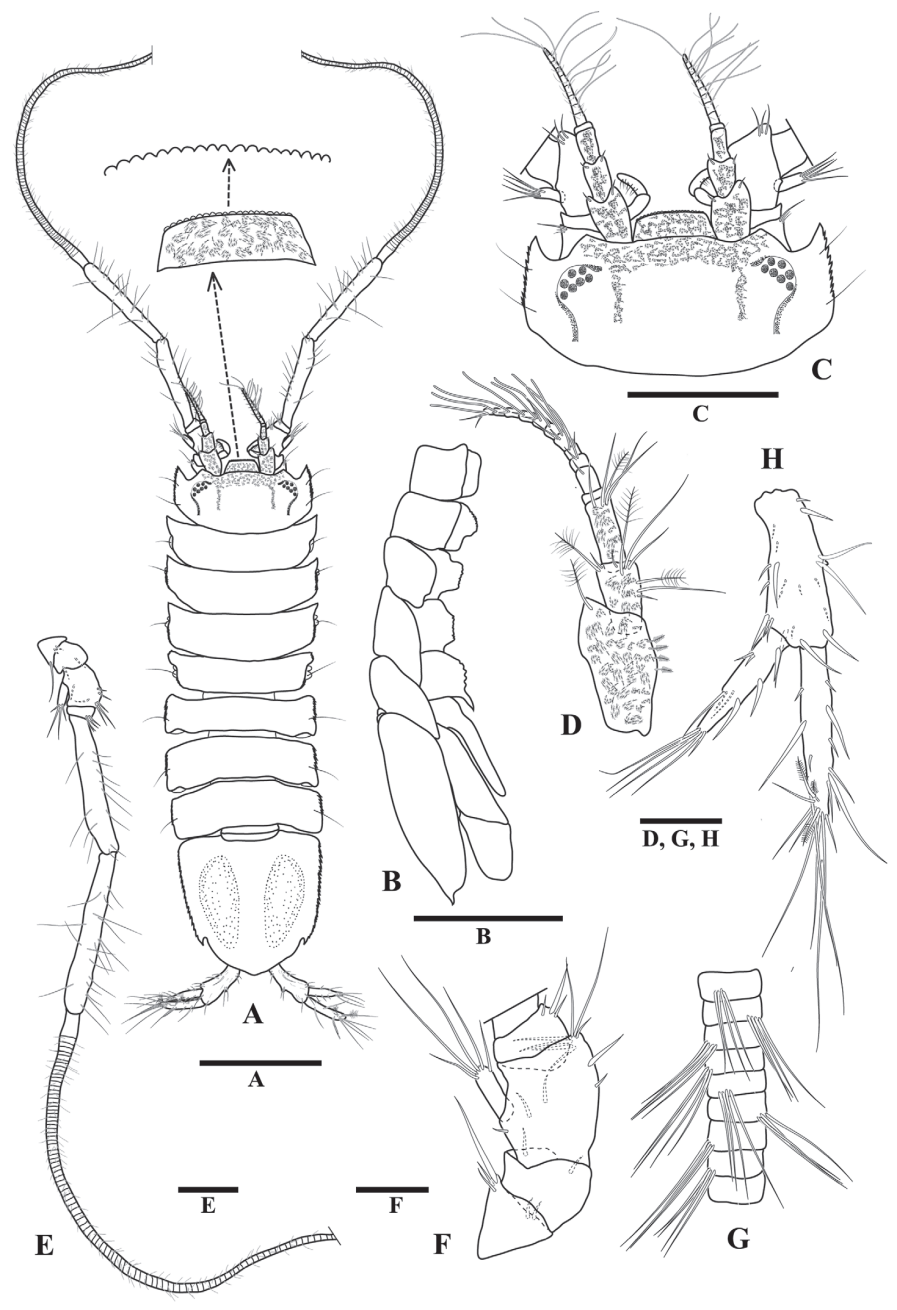
*Tenupedunculus serrulatus* sp. n., male holotype. **A** body, dorsal view **B** body, lateral view, sternal keel **C** cephalon, dorsal view **D** antennula **E** antenna **F** enlargement of peduncular articles 1–4 of antenna **G** enlargement of antennal flagellum articles **H** uropod. Scale bars: 1 mm (**A, B**), 0.5 mm (**C**), 0.2 mm (**D, F, G, H**), 0.4 mm (**E**).

**Figure 2:**
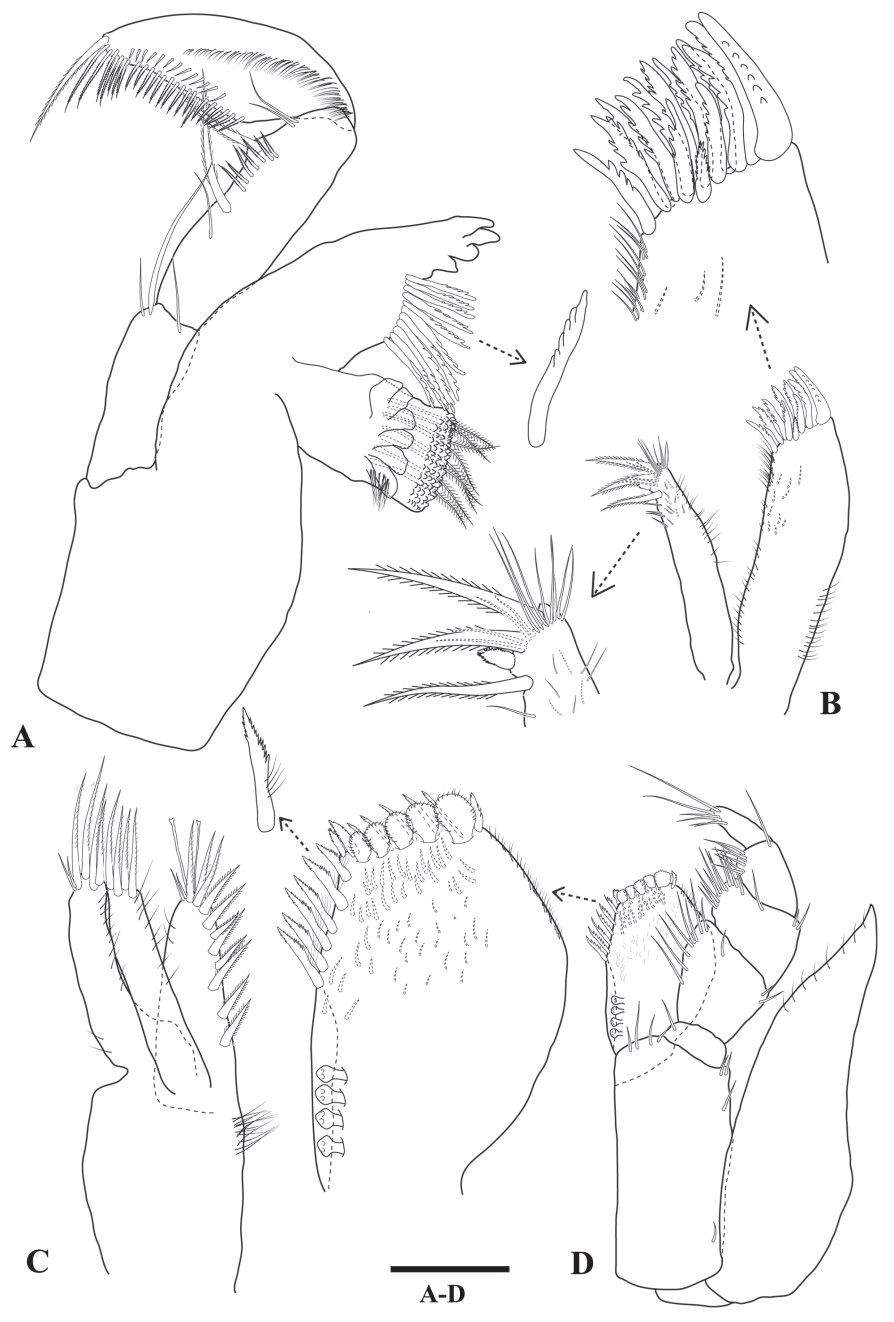
*Tenupedunculus serrulatus* sp. n., male holotype. **A** mandible with palp **B** maxillula, with details of mesial and lateral lobes **C** maxilla **D** maxilliped, with enlargement of endite. Scale bar: 0.1 mm.

**Figure 3:**
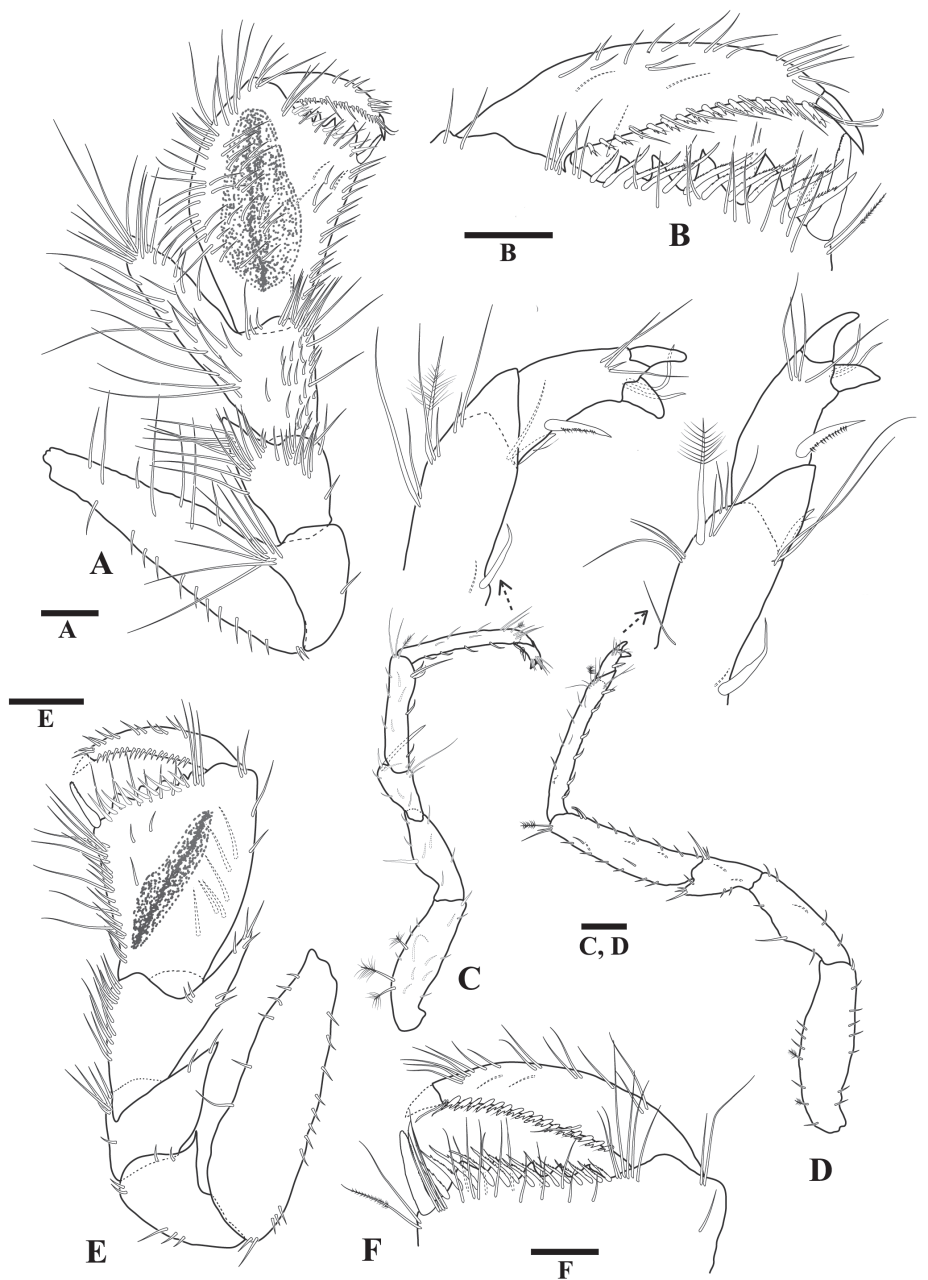
*Tenupedunculus serrulatus* sp. n., **A–D** male holotype **E, F** female paratype. **A** pereopod 1 **B** enlargement of pereopod 1 palm and dactylus **C** pereopod 2 **D** pereopod 7 **E** pereopod 1 **F** enlargement of pereopod 1 palm and dactylus. Scale bars: 0.2 mm (**A, C, D, E**), 0.1 mm (**B, F**).

**Figure 4:**
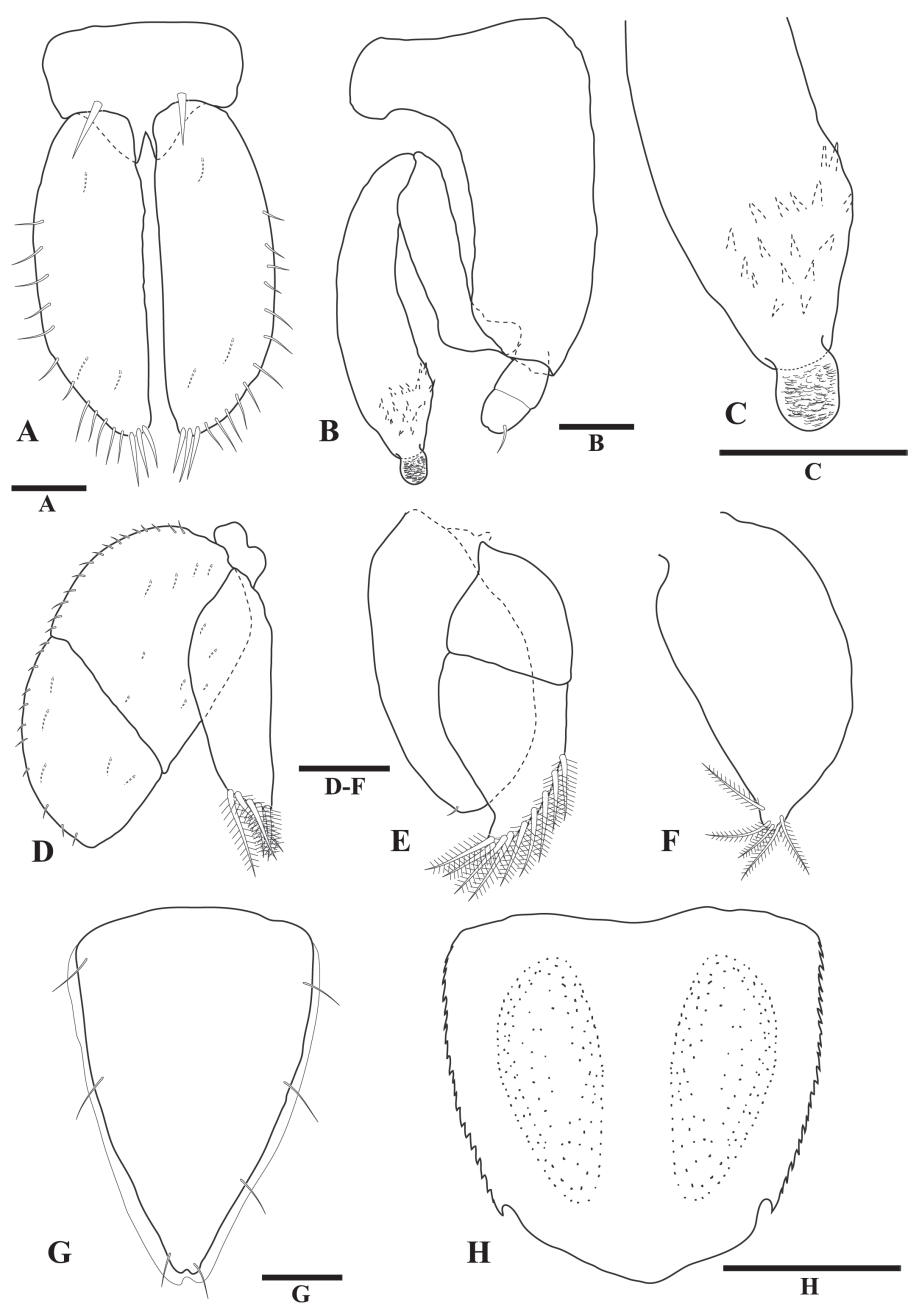
*Tenupedunculus serrulatus* sp. n., **A–F, H** male holotype **G** female paratype. **A** pleopod 1 **B** pleopod 2 **C** enlargement of appendix masculina apex **D** pleopod 3 **E** pleopod 4 **F** pleopod 5 **G** pleopod 2 **H** pleotelson, dorsal view. Scale bars: 0.1 mm (**A, G**), 0.05 mm (**B, C**), 0.2 mm (**D–F**), 0.5 mm (**H**).

**(male).** Body (Fig. 1A) lateral margins with serrations. Pereonite 4 smallest. Pseudorostrum (Fig. 1C) wider than long, trapezoid-shape. Antennula (Fig. 1A, C) longer than cephalon, with ten flagellar articles. Antenna (Fig. 1A) longer than whole body length, with numerous flagellar articles. Maxilliped (Fig. 2D) endite distal margin with five fan setae. Pereopod 1 (Fig. 3A) superior carpal process distinctly long, bladelike. Uropods (Fig. 1A, H) well-developed, biramous, shorter than pleotelson; exopod shorter than endopod. Pleotelson (Fig. 4H) lateral margins with distinct notch.

###### Description

**(male).** *Body* (Fig. 1A) length 3.3 times maximum width. *Cephalon* (Fig. 1C) length 0.5 times width, 1.6 times pereonite 1 length; lateral margins straight or very weakly convex, serrate, with two setae; antennal spines rounded; lateral spines moderate, acute and serrate, longer than antennal spines; space between lateral and antennal spines evenly rounded. *Eyes* (Fig. 1C) with seven ommatidia, pale brown, arranged in circle. *Pereonites 1–7* (Fig. 1A) lateral margin serrate, with one seta. *Sternal keel* (Fig. 1B) present both in males and females as anteriorly directed spines on posteriorly directed spine on pereonites 6. *Pereonite 1* length 0.3 times width, 0.9 times pereonite 2 length, width 1.1 times cephalon width. *Pleotelson* (Figs 1A, 4H) length 0.9 times width; lateral margin serrate, with distinct notch.

*Antennula* (Fig. 1A, D) length 1.5 times cephalon length; article 1 length 1.6 times width, mesial margin with four short penicillate setae, distolateral margin with one large penicillate seta; article 2 length 1.8 times width, distomesial margin with one cluster of setae, including two penicillate seta, distolateral margin with one large penicillate seta and one short seta; article 3 length 2.9 times width, distomesial margin with one cluster of setae; article 4 length 0.3 times width, distomesial margin with one penicillate seta; flagellum with ten articles, one aesthetasc per article on distal nine articles.

*Antenna* (Fig. 1A, E, F, G) length approximately 1.5 times body length; peduncle article 1 length 0.8 times width, distolateral margin with one cluster of setae; article 2 length 0.6 times width, distal margin with two setae; article 3 length 1.1 times width, distomesial margin with two clusters of setae, mesial margin with one long seta and one short seta, lateral margin with five setae surrounding squama; article 4 length 0.7 times width, distomesial margin with three setae; article 5 length 6.8 times width; article 6 length 7.9 times width; each flagellum article with a cluster of four distally projecting setae, the cluster position serially repeating every four articles.

*Mandible* (Fig. 2A) left spine row with eleven spines, right spine row with six spines; palp article 1 length 2.5 times width, distolateral margin with one long seta, and two short setae; palp article 2 length 2.9 times width, with row of seven short serrate setae; article 3 length 2.6 times width. Maxillula (Fig. 2B) lateral lobe apex with 14 serrate RS; mesial lobe apex with two large plumose setae, distomesial margin with one setulate RS, one large plumose seta. Maxilla (Fig. 2C) mesial lobe mesial margin with eight large plumose setae, apex with three large setulate setae, two setae with spatulate tips; middle lobe apex with four large setulate setae; lateral lobe apex with four large setulate setae. Maxilliped (Fig. 2D) basis length 2.2 times maximum width, width 1.1 times endite width; endite distal margin with five fan setae, distomesial margin with six serrate setae, distomesial corner with two triangular RS; epipod length 3.2 times width, width 1.2 times basis width, apex acute, distomesial margin with eight regularly spaced setae, lateral margin sinuate.

*Pereopod 1* (Fig. 3A) basis length 3.7 times width; superior margin with three long setae alternate with three short; submarginal row of short setae.

*Pereopod 1* ischium length 1.6 times width; inferior margin with one short seta; superodistal margin produced, with eight long setae, apex acute.

*Pereopod 1* merus angular; merus length 1.1 times width, 0.8 times carpus length, 0.8 times ischium length; inferior margin with one short seta, two long setae; inferior submargin with a dense patch of long setae; distal margin with no setae; superodistal margin produced, apex acute, densely setose with long setae and two short setae.

*Pereopod 1* carpus triangular; length 1.5 times width, 1.1 times ischium length; distal margin convex, with two short setae; inferior margin clearly defined, and densely setose along distal two-thirds only; inferior submargin with a dense patch of short setae. Superior carpal process long, bladelike; length 3.3 times width, 1.7 times carpal width; extending distally approximately half length of propodus; apex acute, densely setose; inferior margin smooth, straight, densely setose along distal two-thirds only, with several rows of setae; superior margin slightly convex, setose along full length.

*Pereopod 1* propodus robust superiorly with inferior side of article flattened; length 1.5 times maximum width, 3.6 times proximal width, 2.2 times ischium length; inferior margin clearly defined, long, 0.7 times propodus length, 0.6 times superior margin length, densely covered with rows of long and serrate setae and with submarginal row of short setae; superior margin setose, setae regularly spaced. Propodal palm (Fig. 3B) width 0.6 times maximum propodus width, slightly oblique; toothed lobe with four teeth, largest tooth length 3.0 times smallest tooth length; short setae inserting between teeth, cluster of setae at articulation and long setae on mesial surface.

*Pereopod 1* dactylus robust; length 4.8 times width, 1.4 times propodal palm width, 0.9 times propodus distal width (not including process), 0.6 times propodus length; superior margin distal third setose, with regularly spaced setae; distal margin setae regularly spaced along entire length; and more sparsely distributed long setae; mesial surface sparsely setose.

*Pereopod 2* (Fig. 3C) basis superior margin with three penicillate setae; ischium superior margin with one large seta; merus superodistal margin produced with one large RS at apex; carpus superodistal margin with cluster of setae, including one penicillate seta, inferior margin with four flagellated RS (most distal paired with one RS); propodus superodistal margin with cluster of setae, including one penicillate seta, inferior margin with four flagellated RS, inferodistal margin with one flagellated RS.

*Pereopod 7* (Fig. 3D) basis superior margin with two penicillate setae; inferior margin without stiff setae; carpus inferior margin with two flagellated RS; propodus inferior margin with five flagellated RS, inferodistal margin with one flagellated RS.

*Pleopod 1* (Fig. 4A) protopod length 0.7 times width, distal margin with pair of robust setae, surface setae absent; rami lateral margins with regularly spaced setae along distal two-thirds of margin, inferior surface without setae. *Pleopod 2* (Fig. 4B) protopod length 2.6 times medial width, basal lobe width 1.8 times medial width, distal lobe distinctly shorter than exopod, distal lobe blunt; endopod length 0.6 times protopod length, without setae; *appendix masculina* (Fig. 4C) length 1.6 times endopod length, 0.9 times protopod length, widest distally; lateral margin without distal groove; mesial margin without setae; apex convex, depression fringed with scale setae; lateral margin without setae. *Pleopod 3* (Fig. 4D) endopod apex with five plumose setae. *Pleopod 4* (Fig. 4E) exopod apex with nine plumose setae. *Pleopod 5* (Fig. 4F) apex with five plumose setae.

*Uropod* (Fig. 1A, H) length 0.2 times body length, 0.7 times pleotelson length; protopod length 2.4 times width; endopod length 1.1 times protopod length, distal and sub-distal margins with three penicillate setae, distal tip with cluster of elongate setae with maximum length 1.5 times endopod length; exopod length 0.8 times protopod length, 0.7 times endopod length, distal tip with cluster of elongate setae with maximum length 0.9 times exopod length.

###### Description

**(female).** *Pereopod 1* (Fig. 3E) basis length 3.2 times width, superior margin with 12 short setae, inferior margin with six short setae. Ischium superodistal margin produced, apex acute. Merus superodistal margin produced; process apex acute. Propodus length 1.4 times distal (maximum) width, 2.8 times proximal width, 1.6 times ischium length; inferior margin length 0.7 times propodus length, densely setose, with a row of setulate setae and a row of simple setae, setae restricted to distal three-quarters of margin; propodal palm (Fig. 3F) width 0.8 times distal propodus width; straight, slightly oblique, with five teeth, inferior end with single robust flagellated seta, and single robust serrate seta adjacent to first tooth, articulation with 14 long setae. Dactylus length 5.6 times width, 1.3 times palm width, inferior margin with 17 regularly spaced robust flagellated setae, with irregularly distributed long setae.

*Pleopod 2* (Fig. 4G) length 1.5 times maximum width; lateral margins with 2–5 setae; not parallel, narrowing posteriorly; posterolateral margins concave, apex slightly notched, asymmetric, with two setae; inferior surface without setae.

###### Size.

Males 2.6–5.1 mm (mean 3.9 mm, *n* = 6); females 2.3–5.8 mm (mean 3.8 mm, *n* = 24).

###### Remarks.

*Tenupedunculus serrulatus* sp. n. is distinguished from other species of *Tenupedunculus* by the following unique combination characters: merus superodistal margin with strongly produced distally rounded process, more than 3.0 times as long as wide; male pleopod 2 appendix masculina bluntly rounded apically, without apical setae; and the lateral margins of the body (from cephalon to pleotelson) are serrated.

*Tenupedunculus serrulatus* is most similar to the deep-water species *T. pulchrum* (Schultz, 1982) with regard to general external appearance, but is distinguished by the following: small body size (< 6 mm vs. 9 mm for the holotype of *T. pulchrum*), serrations on lateral margin of the whole body (vs. smooth); the very strongly produced process on pereopod 1 carpus superodistal margin, approximately 2.5 times as long as width of carpus proximal margin (vs. weak process, approximately 0.6 times as long as width of carpus proximal margin); and appendix masculina without any acute part (vs. with small acute part). *Tenupedunculus serrulatus* is the only shallow-water tropical species in the genus.

###### Distribution.

Heron Island and reefs of the Capricorn Group, southern Great Barrier Reef, Australia; at depths of 6–27 m.

##### Genus Stenobermuda

Taxon classificationAnimaliaIsopodaStenetriidae

Schultz, 1979

Stenobermuda Schultz, 1979: 905.– Kensley and Schotte 1989: 106.– Serov and Wilson 1995: 77.– Kensley and Schotte 2002: 1456.
Stenetrigus Schultz, 1982: 58.

###### Type species.

*Stenobermuda acutirostrata* Schultz, 1979; by original designation and monotypy.

###### Species included.

*Stenobermuda acutirostrata* Schultz, 1979, Bermuda (type species); *S. brucei* Kensley & Schotte, 2002, Zanzibar, Tanzania; *S. iliffei* Kensley, 1994, Bermuda; *S. mergens* Botosaneanu & Iliffe, 1999, Bahamas; *S. syzygus* (Barnard, 1940), South Africa.

###### Remarks.

*Stenobermuda* is a small genus with both marine and stygobiont species. Recent diagnoses do not require modification in light of other recent revisions, and the genus can be readily recognised by the prominent, narrow, and acute rostral process, small or absent eyes, and pereopod 1 articles without prominent processes or an expanded propodus. Sexual dimorphism is weak in the genus.

A diagnostic character of the genus *Stenobermuda* Schultz, 1979 is the acute and distinct rostrum, but one species, *S. iliffei* Kensley, 1994 is described as having a rostrum but figured with a pseudorostrum. The presence or absence of a rostrum of *S. iliffei* therefore does need to be confirmed to assess its status within the genus. Other than the apparent difference is rostrum the species agrees entirely with *Stenobermuda*.

###### Key to the species of *Stenobermuda*

Cave species are indicated by brackets.

**Table:** 

1	Eyes (ommatidia) absent	[***S. mergens***]
–	Eyes (ommatidia) present	**2**
2	Dorsal coxal plates absent	**3**
–	Dorsal coxal plates present	**4**
3	Body length < 3 mm; pereopod 1 propodus narrow, length 1.9 times maximum width	[***S. iliffei***]
–	Body length > 6 mm; pereopod 1 propodus expanded, length 1.1 times maximum width	***S. syzygus***
4	Eyes with five ommatidia; dorsal coxal plates large	***S. acutirostrata***
–	Eyes with four ommatidia; dorsal coxal plates small	**5**
5	Pereopod 1 propodus expanded, length 1.1 times maximum width; rostrum proximal lateral margin straight	***S. brucei***
–	Pereopod 1 propodus narrow, length 1.9 times maximum width; rostrum proximal lateral margin convex	***S. warooga* sp. n.**

##### Stenobermuda warooga

Taxon classificationAnimaliaIsopodaStenetriidae

Song & Bruce sp. n.

http://zoobank.org/720698E6-C575-4253-97E3-4EFE0D82F482

Figs 5 , 6 , 7 , 8

###### Material examined.

**Holotype.** ♂ (1.6 mm), Yonge Reef, northern Great Barrier Reef, 14.57302°S, 145.6189°E, 10 September 2010, outer reef slope, coarse sand, 25 m, CReefs stn. LI10-126B (MTQ W32968).

###### Paratypes.

3 ♂ (1.7 [all appendage dissected], 1.2 [antennula and pleopod 2 dissected], 1.5 mm [pleopod 1 dissected]), same data as holotype (MTQ W52909). 3 ♂ (1.4, 1.5 [dissected], 1.7 mm), High Rock (between Direction Islands and Ribbon Reef No. 10), northern Great Barrier Reef, 14.82462°S,145.552°E, 6 September 2010, coral rubble, 8 m, CReefs stn. LI10-092A, coll. C. Buxton (MTQ W32917). ♂ (1.2 mm), 14.57302°S, 145.61980°E, Yonge Reef, 10 November 2010, outside; small coral rubble in spur, 20 m CReefs stn LI10-126A (MTQ W52910). ♂ (1.4 mm), Yonge Reef, 14.60681°S, 145.6311°E, 20 February 2009, outer reef front., dead coral, 30 m, coll. CReefs stn LI09-15B Shawn Smith & Julian Caley (MTQ W52911).

###### Etymology.

The epithet ‘*warooga*’ is an Aboriginal word meaning small child, in reference to the small size of this species; noun in apposition.

###### Diagnosis

**Figure 5:**
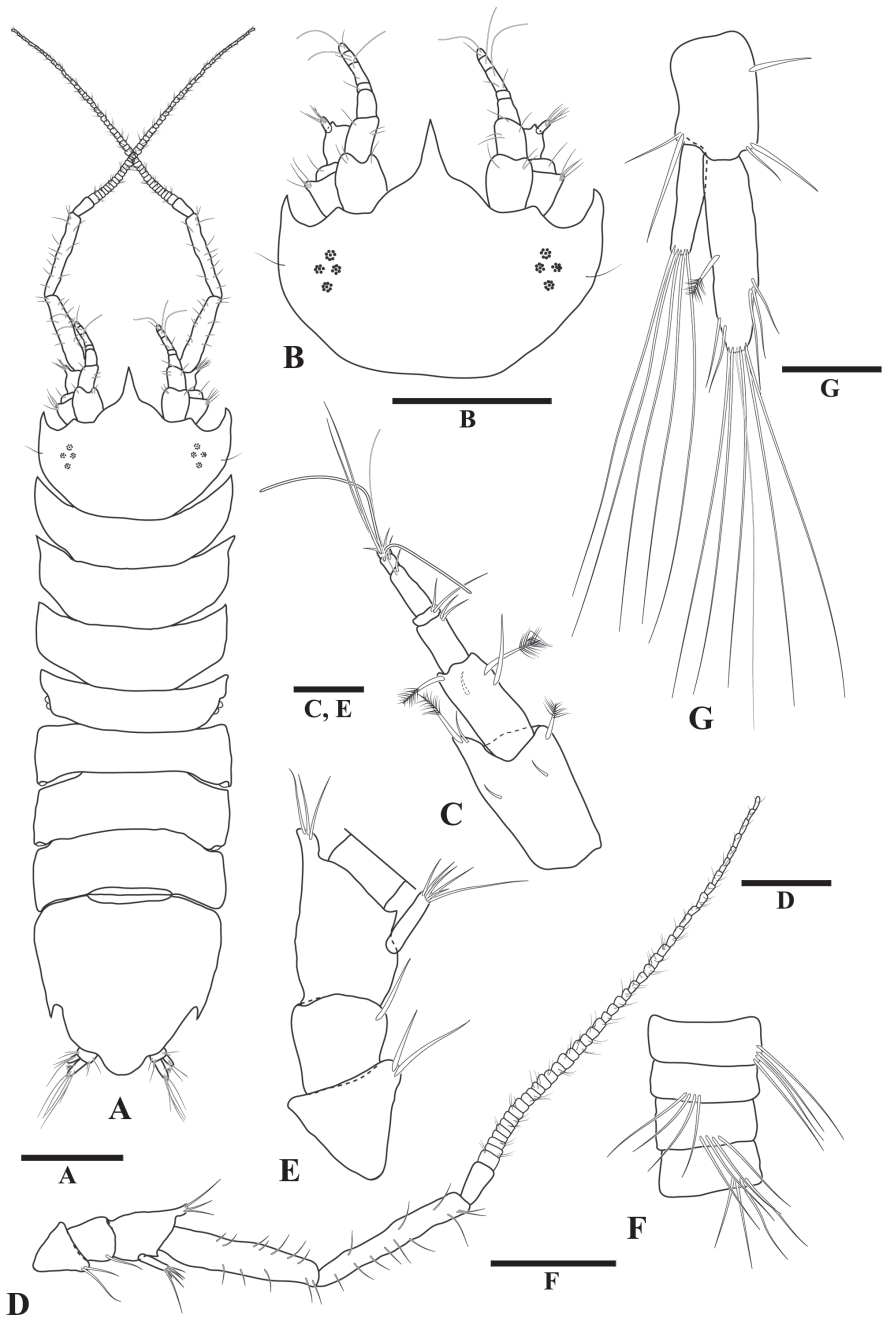
*Stenobermuda warooga* sp. n., male holotype. **A** body, dorsal view **B** cephalon, dorsal view **C** antennula **D** antenna **E** enlargement of peduncular articles 1–4 of antenna **F** enlargement of antennal flagellum articles **G** uropod. Scale bars: 0.25 mm (**A**), 0.125 mm (**B**), 0.05 mm (**C, E, F, G**), 0.2 mm (**D**).

**Figure 6:**
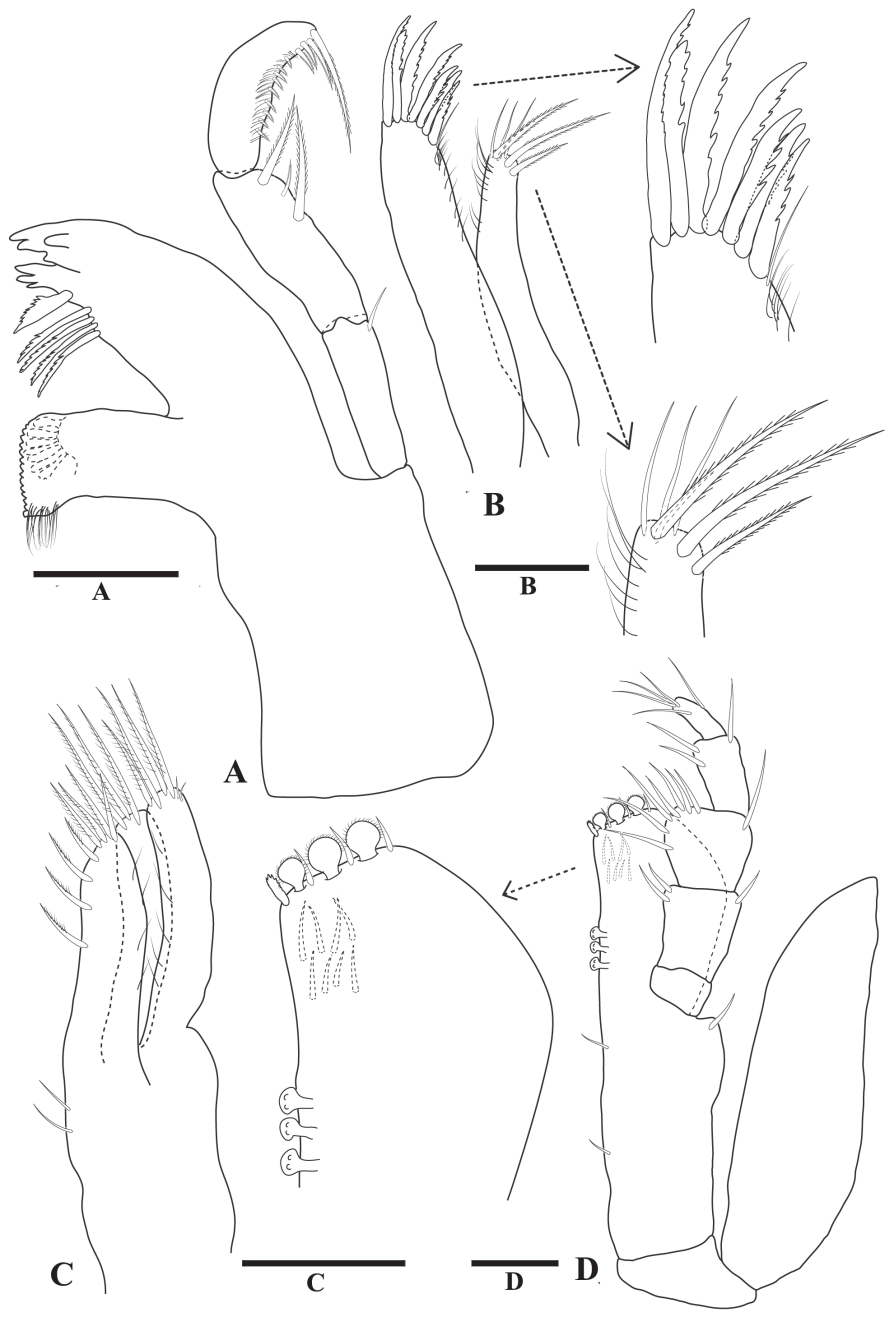
*Stenobermuda warooga* sp. n., male holotype. **A** mandible with palp **B** maxillula, with details of mesial and lateral lobes **C** maxilla **D** maxilliped, with enlargement of endite. Scale bars: 0.05 mm.

**Figure 7:**
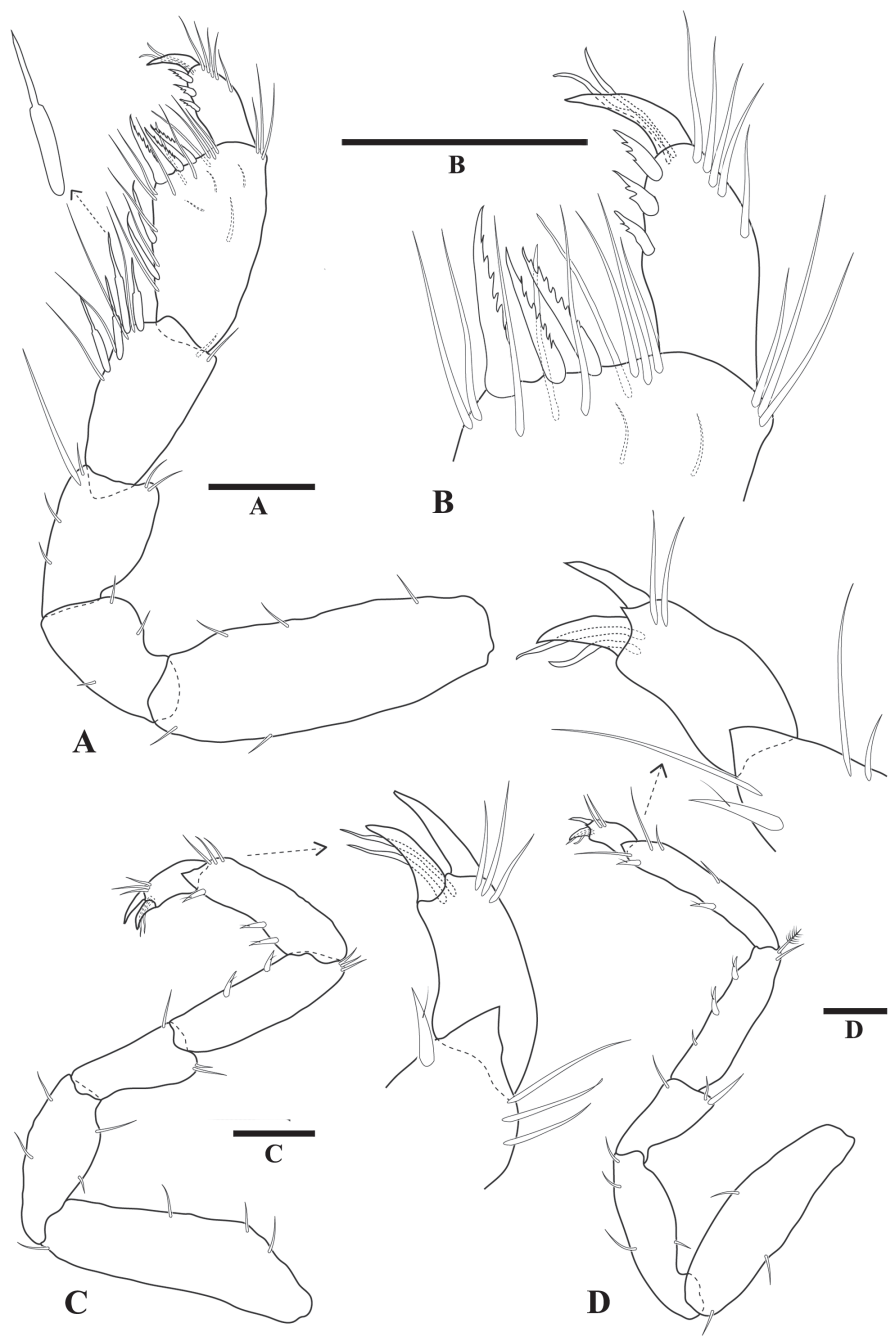
*Stenobermuda warooga* sp. n., male holotype. **A** pereopod 1 **B** enlargement of pereopod 1 palm and dactylus **C** pereopod 2, with enlargement of dactylus **D** pereopod 7, with enlargement of dactylus. Scale bars: 0.05 mm.

**Figure 8:**
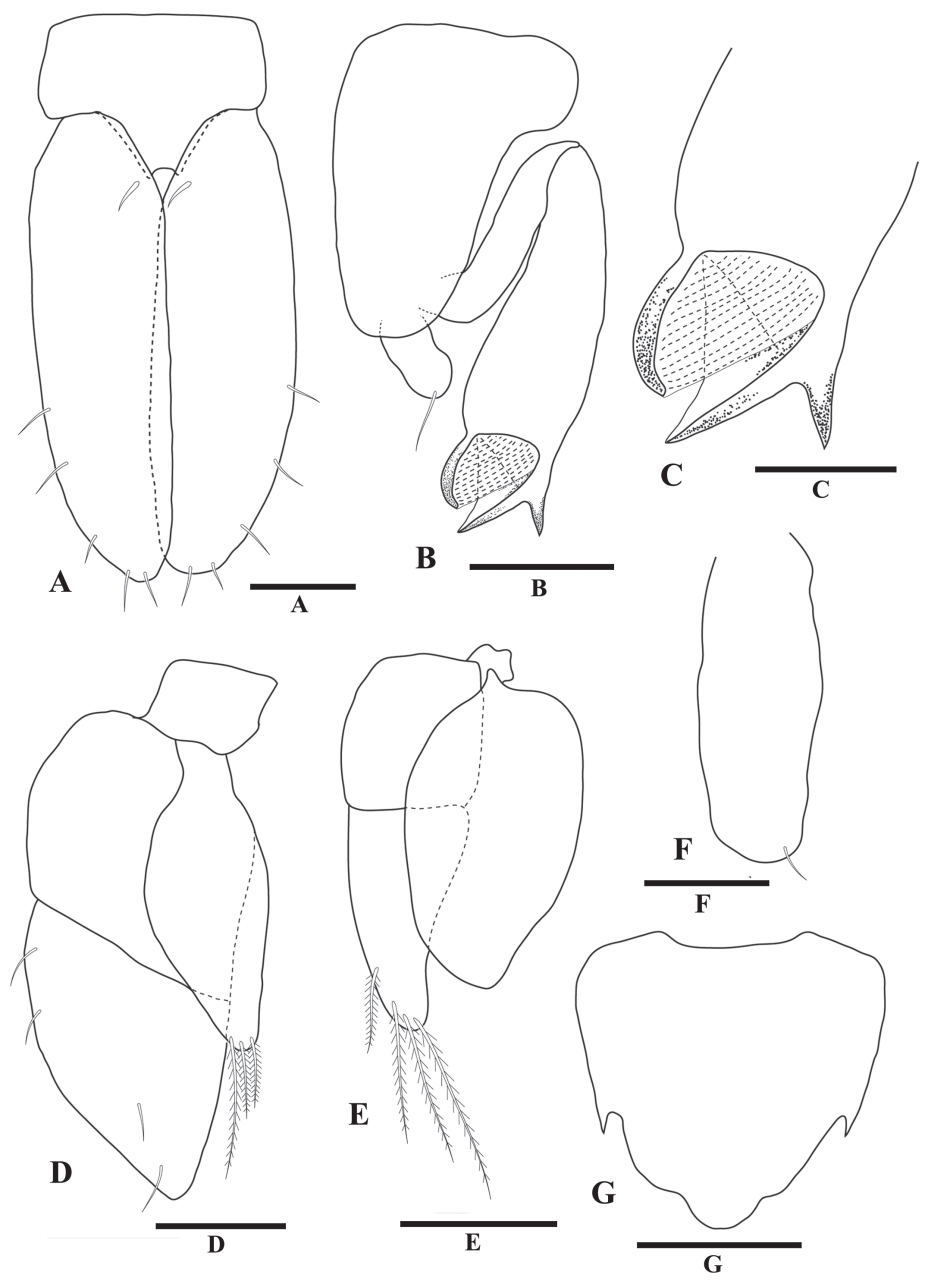
*Stenobermuda warooga* sp. n., male holotype. **A** pleopod 1 **B** pleopod 2 **C** enlargement of appendix masculina apex **D** pleopod 3 **E** pleopod 4 **F** pleopod 5 **G** pleotelson, dorsal view. Scale bars: 0.05 mm (**A, B**), 0.025 mm (**C**), 0.1 mm (**D–F**), 0.25 mm (**G**).

**(male).** Body (Fig. 5A) lateral margins smooth. Pereonite 4 smallest. Rostrum (Fig. 5B) acute, proximal lateral margin convex. Antennula (Fig. 5C) shorter than cephalon, with three flagellar articles. Antenna (Fig. 5D) shorter than whole body length, with numerous flagellar articles. Maxilliped (Fig. 6D) endite distal margin with three fan setae. Pereopod 1 (Fig. 7A) superodistal and inferodistal margin without process. Uropod (Fig. 5A, G) very short, biramous; exopod shorter than endopod. Pleotelson (Fig. 8G) lateral margins with distinct notch.

###### Description

**(male).** *Body* (Fig. 5A) length 3.6 times maximum width. *Cephalon* (Fig. 5B) length 0.8 times width, 3.2 times pereonite 1 length; lateral margins straight or very weakly convex, smooth, with one setae; antennal spines acute; lateral spines acute, longer than antennal spines. *Rostrum* (Fig. 5B) proximal lateral margin convex. *Eyes* (Fig. 5B) with four ommatidia, pale brown, arranged in circle. *Pereonites 1–7* (Fig. 5A) lateral margin smooth, without setae; pereonite 1 length 0.2 times width, 0.8 times pereonite 2 length, width 1.1 times cephalon width; pereonite 5–7 distolateral margin not produced. *Coxal plates* (Fig. 5A) small, visible dorsally on pereonites 4–6. *Pleotelson* (Figs 5A, 8G) length 0.9 times width, with distinct notch.

*Antennula* (Fig. 5C) length 0.7 times cephalon length; article 1 length 1.6 times width, mesial margin with one short penicillate setae, distolateral margin with one large penicillate seta; article 2 length 2.3 times width, distolateral margin with one large penicillate seta; article 4 length 0.5 times width; flagellum with three articles, one aesthetasc per article on distal two articles.

*Antenna* (Fig. 5D, E, F) length approximately 0.7 times body length; peduncle article 1 length 0.8 times width; article 2 length 0.9 times width, distolateral margin with one long seta; article 3 length 1.3 times width, distomesial margin with one cluster of setae, lateral margin with six setae surrounding squama; each flagellum article with a cluster of four distally projecting setae, the cluster position serially repeating every four articles.

*Mandible* (Fig. 6A) left spine row with four spines, right spine row with six spines; palp article 1 length 3.5 times width, distolateral margin with one long seta; palp article 2 length 2.7 times width, with row of one short serrate setae; article 3 length 2.8 times width. *Maxillula* (Fig. 6B) lateral lobe apex with seven serrate RS; mesial lobe apex with three large plumose setae. *Maxilla* (Fig. 6C) mesial lobe mesial margin with four large plumose setae, apex with four large setulate setae; middle lobe apex with three large setulate setae; lateral lobe apex with four large setulate setae. *Maxilliped* (Fig. 6D) basis length 2.0 times maximum width, width 0.9 times endite width; endite distal margin with three fan setae, distomesial corner with one triangular RS; epipod length 3.4 times width, width 1.1 times basis width, apex acute, lateral margin evenly convex.

*Pereopod 1* (Fig. 7A) basis length 3.2 times width; superior margin with three short setae; submarginal row of short setae.

*Pereopod 1* ischium length 1.9 times width; inferior margin with one short seta; distal margin with one short setae; superodistal margin not produced, apex rounded.

*Pereopod 1* merus rectangular; merus length 1.4 times width, 0.8 times carpus length, 0.9 times ischium length; inferior margin with two short setae, one long seta; distal margin with no setae; superodistal margin not produced, apex rounded, with two short setae.

*Pereopod 1* carpus rectangular; length 2.0 times width, 1.3 times ischium length; distal margin convex; inferior margin clearly defined, and with four stout setae and four long setae; superodistal margin not produced, apex obliquely truncate.

*Pereopod 1* propodus robust and narrow; length 1.9 times maximum width, 3.2 times proximal width, 1.3 times ischium length; inferior margin clearly defined, long, 0.7 times propodus length, 0.7 times superior margin length, lightly setose, regularly spaced setae along entire length; superior margin setae absent. Propodal palm (Fig. 7B) width 0.7 times maximum propodus width, slightly oblique; with three large serrate setae and nine long setae. Pereopod 1 dactylus narrow.

*Pereopod 1* dactylus convex in mid-section; length 1.9 times width, 1.3 times propodal palm width, 0.8 times propodus distal width (not including process), 0.4 times propodus length; superior margin distal third with four long setae. Distal margin with three serrate setae. Mesial surface not setose.

*Pereopod 2* (Fig. 7C) basis medial inferior margin with stiff seta absent; ischium superior margin with stiff setae absent; merus superodistal margin RS absent; carpus superodistal margin with three setae, inferior margin with two flagellated RS (most distal paired with one RS); propodus superodistal margin with three setae, inferior margin with two flagellated RS, inferodistal margin with one flagellated RS.

*Pereopod 7* (Fig. 7D) basis inferior margin without stiff setae; carpus inferior margin with two flagellated RS; propodus inferior margin with one flagellated RS, inferodistal margin with one flagellated RS.

*Pleopod 1* (Fig. 8A) protopod length 0.7 times width, surface setae present; rami lateral margins with regularly spaced setae along distal half, inferior surface without setae. *Pleopod 2* (Fig. 8B) protopod length 1.9 times medial width, basal lobe width 1.4 times medial width, distal lobe distinctly shorter than exopod, distal lobe blunt; endopod length 0.7 times protopod length, without setae; *appendix masculina* (Fig. 8C) length 1.8 times endopod length, 1.3 times protopod length, widest distally; lateral margin without distal groove; mesial margin without setae; apex with two process and cuticular fan; lateral margin without setae. *Pleopod 3* (Fig. 8D) endopod apex with three plumose setae. *Pleopod 4* (Fig. 8E) exopod apex with four plumose setae. *Pleopod 5* (Fig. 8F) apex with one seta.

*Uropod* (Fig. 5A, G) very short, length 0.05 times body length, 0.2 times pleotelson length; protopod length 1.4 times width; endopod length 1.5 times protopod length, distal and sub-distal margins with one penicillate setae, distal tip with cluster of elongate setae with maximum length 1.9 times endopod length; exopod length 0.9 times protopod length, 0.6 times endopod length, distal tip with cluster of elongate setae with maximum length 3.6 times exopod length.

###### Female.

Not known.

###### Size.

Males 1.2–1.7 mm (mean 1.5 mm, *n* = 9).

###### Remarks.

*Stenobermuda warooga* sp. n. can be identified by the following unique combination characters: small body size of the adult male (< 2 mm); rostrum proximal lateral margin with convex margin; pereonite 5 distolateral margin not produced; pereopod 1 propodus narrow, length 1.9 times maximum width (Table 2). The most similar species is *S. brucei* Kensley & Schotte, 2002, a species also occurring on coral reefs, with regard to external appearance. However, propodus of pereopod 1 is strongly expanded, length 1.1 times maximum width, and proximal lateral margin of rostrum is straight in *S. brucei* (propodus of pereopod 1 is narrow, length 1.9 times maximum width, and proximal lateral margin of rostrum is convex in *S. warooga* sp. n.).

**Table 2.** Comparison of diagnostic characters between *S. warooga* sp. n. and other species of *Stenobermuda* (male).

	**Total length (mm) of adult male**	**Rostrum (Pseudorostrum), proximal lateral margin**	**Rostrum/ Pseudorostrum**	**Eyes (ommatidia)**	**Dorsally visible coxae (size)**	**Pereopod 1 propodus length**
***S. warooga* sp. n.**	**1.6**	**Convex**	**Rostrum**	**Small rounded (4 ommatidia)**	**Pereonites 4–6 (small)**	**Narrow, 1.9 times maximum width**
*S. acutirostrata*	4.8	Straight	Rostrum	Small rounded (5 ommatidia)	Pereonites 1, 4–6 (large)	Normal, 1.6 times maximum width
*S. brucei*	3.1	Straight	Rostrum	Small rounded (4 ommatidia)	Pereonites 4–6 (small)	Expanded, 1.1 times maximum width
*S. iliffei*	2.9	Straight	Pseudorostrum (?)	Small rounded (4 ommatidia)	Not visible	Narrow, 1.9 times maximum width
*S. mergens*	3.2	Straight	Rostrum	Absent	Pereonites 1–6 (small)	Normal, 1.6 times maximum width
*S. syzygus*	6.5	Straight	Rostrum	Small rounded (4 ommatidia)	Not visible	Expanded, 1.1 times maximum width

###### Distribution.

Yonge Reef and High Rock, Lizard Island region, northern Great Barrier Reef both outer barrier reefs; at depths of 8–30 m.

## Supplementary Material

XML Treatment for Tenupedunculus

XML Treatment for Tenupedunculus serrulatus

XML Treatment for Stenobermuda

XML Treatment for Stenobermuda warooga
